# The *Drosophila* tracheal terminal cell as a model for branching morphogenesis

**DOI:** 10.1073/pnas.2404462121

**Published:** 2024-10-02

**Authors:** Tatyana Gavrilchenko, Alison G. Simpkins, Tanner Simpson, Lena A. Barrett, Pauline Hansen, Stanislav Y. Shvartsman, Jodi Schottenfeld-Roames

**Affiliations:** ^a^Flatiron Institute, Simons Foundation, New York, NY 10010; ^b^Lewis-Sigler Institute for Integrative Genomics, Princeton University, Princeton, NJ 08544; ^c^Department of Molecular Biology, Princeton University, Princeton, NJ 08544; ^d^McKinsey & Company, Philadelphia, PA 19104

**Keywords:** insect trachea, terminal cell, morphogenesis, network, scaling laws

## Abstract

The terminal cells of the *Drosophila* larval tracheal system are perhaps the simplest delivery networks, providing an analogue for mammalian vascular growth and function in a system with many fewer components. These cells are a prime example of single-cell morphogenesis, branching significantly over time to adapt to the needs of the growing tissue they supply. While the genetic mechanisms governing local branching decisions have been studied extensively, an understanding of the emergence of a global network architecture is still lacking. Mapping out the full network architecture of populations of terminal cells at different developmental times of *Drosophila* larvae, we find that cell growth follows scaling laws relating the total edge length, supply area, and branch density. Using time-lapse imaging of individual terminal cells, we identify that the cells grow in three ways: by extending branches, by the side budding of new branches, and by internally growing existing branches. A generative model based on these modes of growth recapitulates statistical properties of the terminal cell network data. These results suggest that the scaling laws arise from the coupled contributions of branching and internal growth. This study establishes the terminal cell as a uniquely tractable model system for further studies of transportation and distribution networks.

## Significance

Multicellular biological distribution systems, such as animal vasculature and leaf venation, have been studied in a wide range of contexts. Here, we study the terminal cells of insect trachea, a model system that provides a powerful paradigm for the quantitative analysis of single-cellular transport networks. A combination of longitudinal and cross-sectional studies of these networks reveals scaling laws that characterize terminal cell development. We determine the macroscopic growth rules and construct a minimal model of a growing network based on these rules, finding that it recovers the observed scaling laws. The scaling analysis provides a compact language for dynamic growing networks. Because of their simplicity and tractability, tracheal terminal cells are an informative model for more complex distribution networks.

Nature solves transport problems with networks. Biological networks in the form of interconnected tubular structures are found in almost all organisms, functioning in the collection and distribution of materials necessary for life. Through recent advances in imaging, it has become possible to resolve the finest details of these biological networks at different developmental times, tracking the progression of a growing network from nascence to maturity. The developmental program of distribution networks varies widely among different organs and organisms. For instance, studies of the mouse bronchial tree have revealed a remarkably stereotyped structure, encoded by a modular genetic program (1). On the other hand, many biological networks develop with some level of stochasticity. For organ systems such as the mouse mammary, salivary, and lymph glands, realistic branching network geometries can be reproduced through branching and self-avoiding random walks (2–4). Similar rules are seen in moss colonies and root systems (5). Growth driven by external signaling appears in diverse systems such as the zebrafish vasculature (6), leaf vasculature (7), and insect gills (8). In cases such as the kidney duct network, additional rules such as self-avoidance through inhibitory signaling are necessary to recapitulate realistic geometries (9).

Insect respiratory networks present an interesting case because they consist of a stereotyped large-scale structure, followed by finer stochastic elements (10). Like other insects, *Drosophila melanogaster* larvae distribute oxygen throughout their bodies with a hierarchal network of intertwined hollow trachea. The finest levels of trachea are called terminal cells and are the components where the majority of gas exchange takes place (11). Each terminal cell has a unique branched shape, forming a binary tree structurally reminiscent of neuronal dendrites. The internal structure of a terminal cell branch is an air-filled lumen encased in cytoplasm, and air passively diffuses from the lumen along the length of the branch into the surrounding tissue. The *Drosophila* tracheal system has received much attention as it is genetically and mechanistically similar to mammalian lung development and vascular formation (12, 13). Because the specialized terminal cell is a single cell with a seamless tube geometry, it is an experimentally tractable and minimally complex model for an organ network composed of several cell populations (14, 15).

**Fig. 1.**
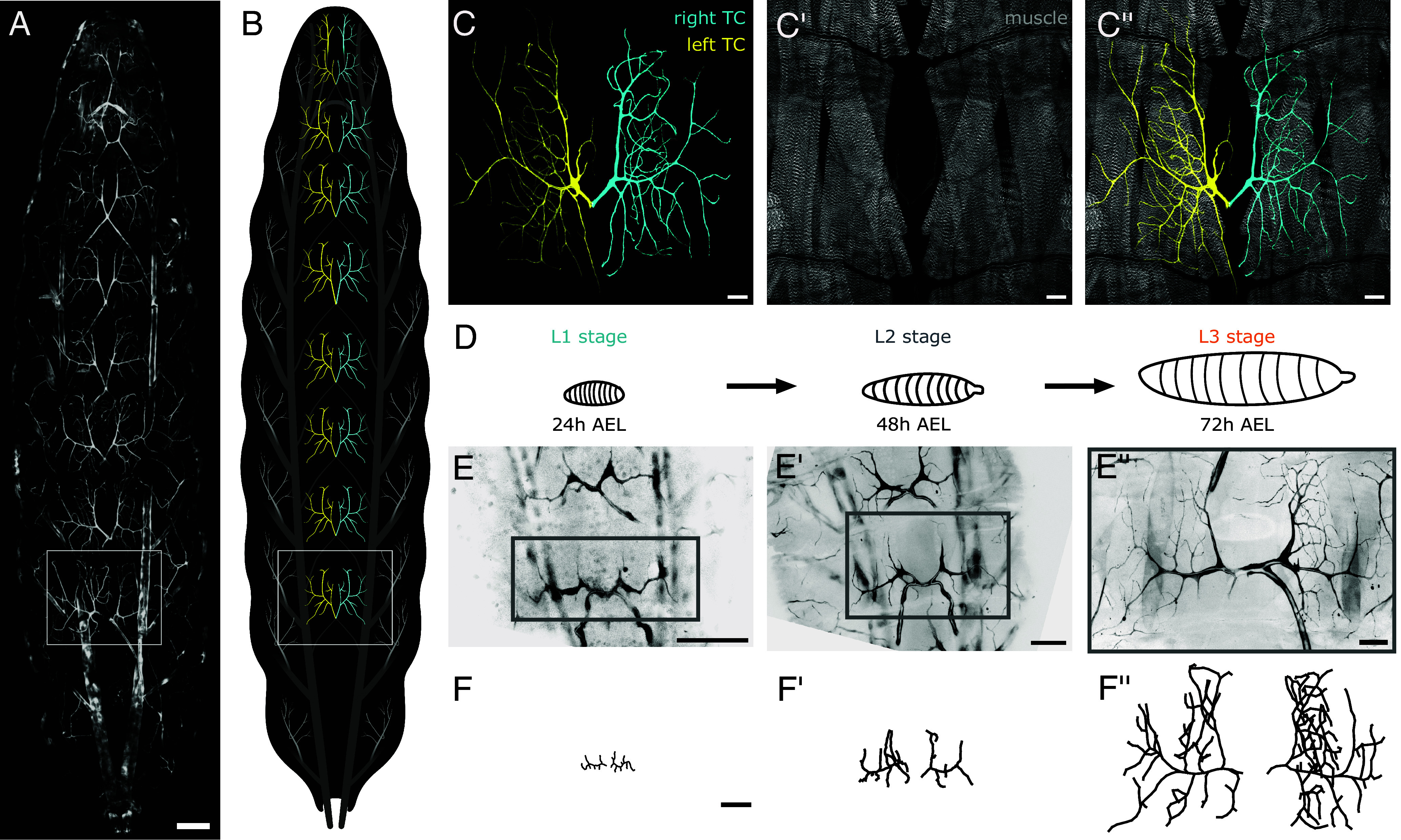
Terminal cells grow significantly over three larval stages. (*A*) The *Drosophila* tracheal system is a hierarchal network of tubes that spans the larval body (cytoplasmic GFP shown in white is driven by the Gal4-UAS under a *breathless* (*btl*) promoter). Eight pairs of terminal tracheal cells are found on the dorsal side of the body; we focus our studies on the posterior-most terminal cells, known as tracheal metamere 9 (Tr9). (*B*) Schematic of the tracheal system, with dorsal terminal cells shown in color and other trachea shown in gray. (*C*) Tracheal terminal cells from larvae heterozygous for btl > gal4, UAS > cytoplasmic reporter at the wandering L3 stage. Dorsal terminal cells appear in *Left*–*Right* pairs (cytoplasmic RFP in yellow and cyan, colored separately to distinguish the two cells). (*C*^′^) Each terminal cell supplies two pairs of body wall muscles (Zasp:GFP in gray). (*C*^″^) Merge of (*C* and *C*^′^). (*D*) Larvae are heat-killed at three different stages: L1, L2, and L3, where the collection time is 24, 48, and 72 h after egg lay, respectively. (*E*–*E*^″^ and *F*–*F*^″^) Tr9 terminal cells images are segmented and the network structure is extracted. (Scale bar in *A* is set to 200 μm; all other scale bars are set to 50 μm.)

The *Drosophila* larva contains roughly 230 terminal cells which are particularly densely branched in metabolically active tissue such as the heart and gut, and in the adult flies, the flight muscles (16, 17). We consider the network structure of the dorsal tracheal terminal cells, found in eight *Left*–*Right* pairs along the back of the animal, supplying the body wall muscles (Fig. 1 *A* and *B*^″^). The terminal cells display some level of self-avoidance, typically occupying nonoverlapping regions on the body wall muscles (17, 18). The branching patterns are highly variable, both among animals and between the *Left* and *Right* sides of an individual. Terminal cells are specified during late embryogenesis, initially appearing as single elongated cells guided by local signaling molecules (19–22). Over the larval lifetime, the larval body size increases by about a factor of five and the terminal cells grow dramatically from simple protrusions into intricate antler-like shapes (Fig. 1*E*–*E*^″^) (20, 23). During this time, terminal cell growth is driven by Fibroblast Growth Factor (Branchless) signal secreted by the surrounding tissues (24, 25). The formation of new branches involves the localization of cytoskeletal components and multicomponent complexes acting both at the external plasma membrane and at the internal lumen, and how the locations are chosen for new branches is not known (26).

In this paper, we study the structure and development of larval tracheal terminal cells using scaling analysis. This method has been used to identify network properties that are conserved across many instances of individual networks of different physical sizes, both in living (27) and nonliving systems (28). Scaling laws relate important structural features of networks, and a robust scaling relation may reveal underlying properties essential to network function (29). At each developmental snapshot, the terminal cell network fulfills the metabolic requirements of the system, so it is reasonable to expect that growth dynamics, encapsulated by the scaling laws, are a critical functional feature. Our goal is to describe the dynamics of network growth in a compact way, allowing us to identify the salient features of network growth and verify potential growth models.

Using the language of network science to quantify cellular structure, we find that the system obeys several empirical scaling laws over the course of development. Based on time-lapse images of individual cells, we formulate a set of growth behaviors of these cells, identifying that the governing features are branching and scaling rules. Given these growth rules, we ask what is the space of possible scaling exponents that the system may attain in order to determine the significance of the observed terminal cell scaling laws. Using intuition from simple toy models of growth, we describe the asymptotic regimes of branching and scaling growth. Finally, we present a generative branching network algorithm and find a parameter range that captures the observed scaling behavior. Surprisingly, the rules that govern terminal cells are similar to rules obeyed in more complex multicellular systems.

## Results

### Terminal Cells Grow Extensively throughout Larval Development.

We first present a cross-sectional dataset of fully resolved pairs of dorsal terminal cells at three different developmental stages. Collection times are chosen to represent each of the larval stages: immediately after hatching in the first instar (L1, 24 h after egg laying [AEL]), immediately after the first molt in the second instar (L2, 48 h AEL), and immediately after the second molt in the third instar (L3, 72 h AEL). In L1 the terminal cells consist of a single lateral branch with several secondary branches, in L2 the terminal cells attain their rudimentary structure, and by L3 the terminal cells are mature (Fig. 1*E*–*E*^″^). The final dataset consists of 52 terminal cells at the L1 stage, 54 at the L2 stage, and 56 at the L3 stage, where cells appear in *Left*–*Right* pairs (*SI Appendix*, Fig. 1).

**Fig. 2.**
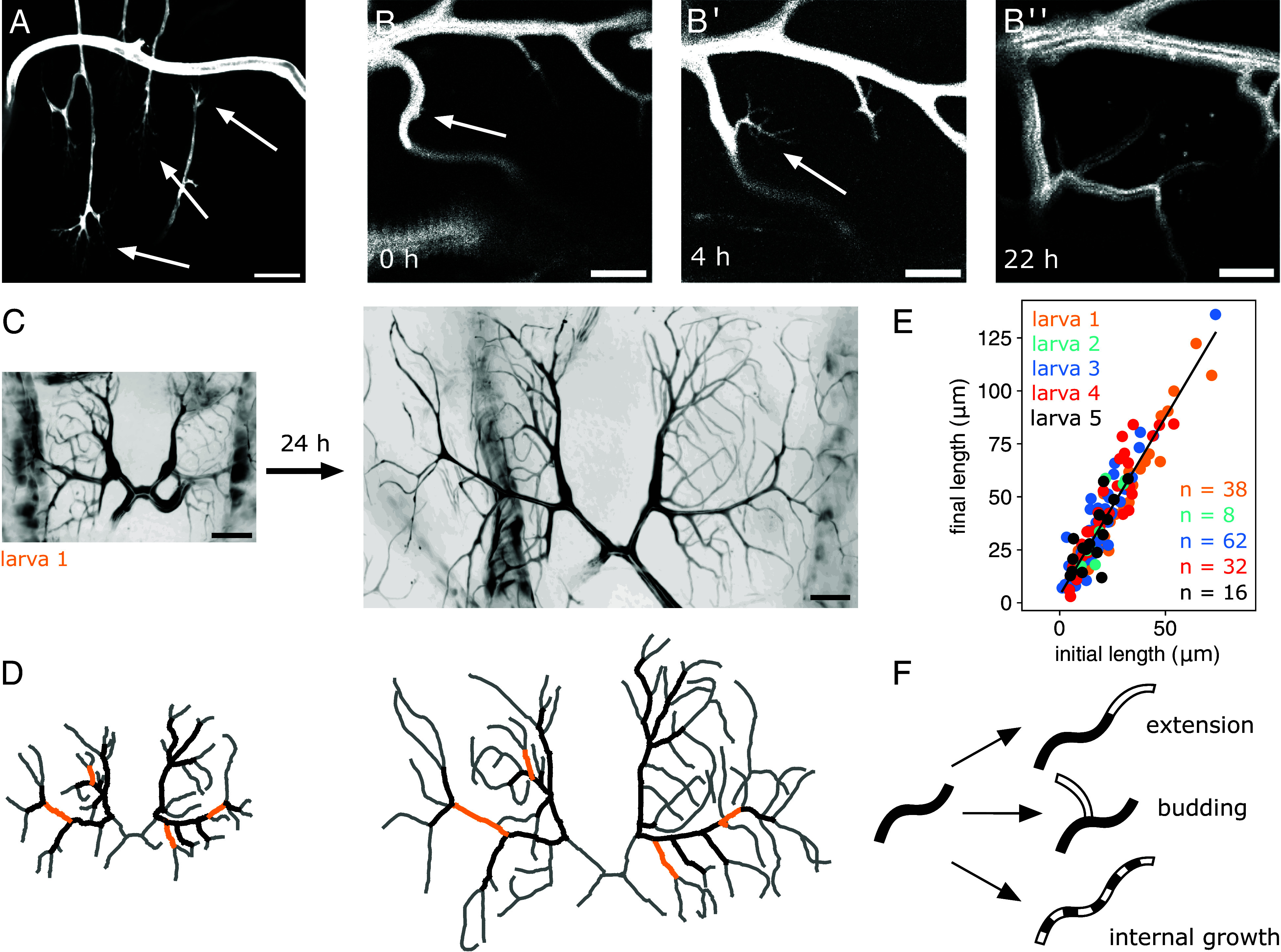
Identifying terminal cell growth rules. (*A*) Filopodial extensions (white arrows) appear at branch tips, guiding branch extension. (Scale bar: 25 μm.) (*B*–*B*^″^) Time-lapse imaging of a terminal cell section in the L1 and L2 stages captures branch budding (white arrows). The branch has no identifiable budding at 0 h, then a distinct filopodium at 4 h, and by 22 h it has grown into a full branch. (Scale bar: 10 μm.) (*C* and *D*) Time-lapse imaging and network tracing of a pair of terminal cells imaged once in the late L2 stage and again 24 h later in the L3 stage. Internal edges present in both stages are shown in black, and four individual internal edges are highlighted as examples. (Scale bar: 25 μm.) (*E*) The time-lapse experiments were repeated on pairs of terminal cells from five different larvae, and between 8 and 62 internal edges were identified per sample. The initial and final lengths of the internal branches were measured from micrograph traces. We found a linear relation between the final and initial branch lengths, with a slope of 1.68±0.05 ($r^{2}=0.88$). (*F*) A summary of the inferred branch growth rules.

Terminal cell network architectures were reconstructed in three dimensions via manual tracing (Fig. 1*F*–*F*^″^) (*Materials and Methods*). The number of network branches increases over the course of development, from 10±3 in L1 to 27±10 in L2 to 95±22 in L3 (*SI Appendix*, Fig. 2). The area spanned by the terminal cell increases as well. Taking the x-dimension to be along the lateral body axis, the y-dimension to be along the anterior-posterior axis, and the the z-dimension to be the depth into the body, the average terminal cell dimensions are (44±8,34±8,3±1) for L1, (103±21,113±23,8±4) for L2, and (244±39,322±48,19±4) for L3, measured in μm (*SI Appendix*, Fig. 2). Thus, terminal cells remain mostly flat across the larval stages, with a depth that is 10 to 15% percent of anterior–posterior dimensions. Because the terminal cells are approximately flat structures, the full network architectures were projected onto the best-fit plane for all network analysis. In these two-dimensional networks, edge overlaps are understood to be projected crossings, not true loops.

It is clear that over the larval stages, terminal cells undergo a morphologic transformation, changing in both topology by increasing in number of branches and in geometry by increasing spatial coverage. Our next goal was to determine the set of rules that act on a network and change its structure.

### Terminal Cell Growth Modes: Branch Extension, Budding, and Internal Growth.

Much work has been done on identifying factors required for terminal growth branching morphogenesis (30–33). However, the macroscopic mechanisms of terminal cell growth, such as the location and timing of new branch formation, are not understood. Membrane maintenance is an active process, as material is constantly being added and removed from the terminal cell apical and basal membranes through endocytosis and exocytosis (34–36). Areas of active branching growth are identifiable by filopodia, thin actin-rich protrusions that are often seen at the leading edge of migratory cells. In terminal cells, filopodial extensions are dynamic; they can grow and retract, but eventually become stabilized with an internal lumen to form a mature branch (37). Filopodia are primarily observed on branch tips (Fig. 2*A*), serving the same purpose as the filopodia of tip cells leading an angiogenic sprout, guiding the direction of the growing vessel (38). In addition, through time-lapse imaging, we observed a filopodium forming in the middle of an established branch and gradually growing into a new branch (Fig. 2*B*). We refer to this process as branch budding. Previous work suggests that branch budding and branch extension are distinct processes requiring different types of chemical complexes (39). Unlike other types of networks, tip bifurcation does not seem to play a role in the construction of these binary trees. Thus, in the terminal cell system, existing branches increase in length through extension, and new branches are added to the system through budding.

**Fig. 3.**
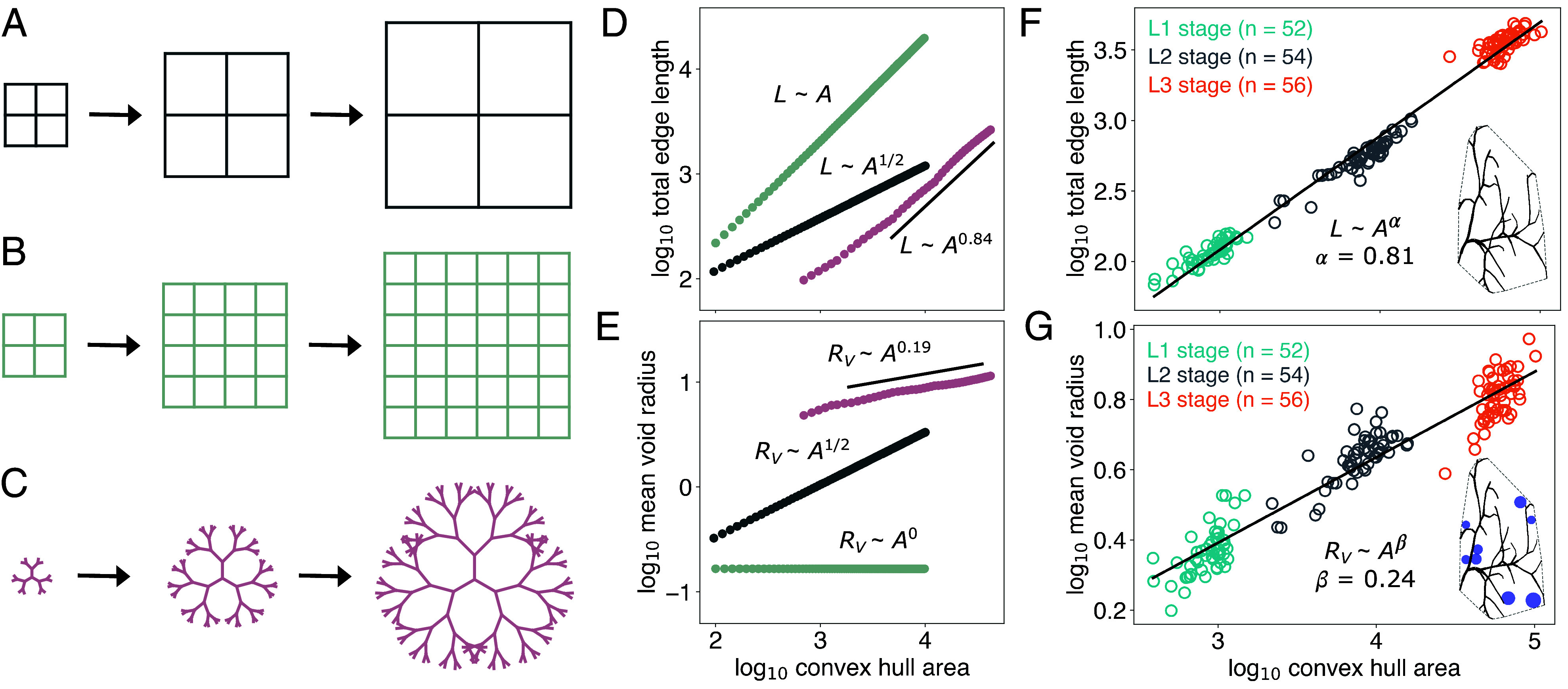
Growth dynamics are encoded in scaling laws, and terminal cells exhibit nontrivial scaling. The two asymptotic growth modes for a square network are (*A*) the scaling mode, and (*B*) the branching mode. (*C*) An example of mixed-mode growth is the Cayley tree where edges continue to lengthen even after bifurcation. (*D* and *E*) The three examples exhibit distinct growth dynamics, as reflected in the scaling relations $L∼A^{\alpha }$ and $R_{v}∼A^{\beta }$. For the two pure modes, scaling exponents can be derived analytically: α=β=0.5 for the scaling mode and α≈1.0 and β=0 for the branching mode. For the Cayley tree, the exponents are numerically found to be α=0.84±0.01 and β=0.190±0.003, attaining intermediate values between the two pure modes. (*F* and *G*) Obtaining measurements for the terminal cell data over three larval stages yields α=0.81±0.01 ($r^{2}=0.975$) and β=0.24±0.01 ($r^{2}=0.847$), indicating that the terminal cells follow a combination of the scaling and branching modes.

We also identified a third mode of growth, where new material is added not at the tip, but along the entire length of the branch. We refer to this mode as internal growth. To demonstrate that internal growth contributes significantly to network growth, we compare the network structures of the same terminal cell imaged at two time points, once in the L2 stage, and a second time 24 h later, when the larva is in the L3 stage (Fig. 2*C*). Here, tracheal metamere 8 is used, but we expect the results to generalize to all dorsal terminal cells. By matching the network structure of the L2 and L3 stage terminal cells, we identify internal edges that appear at both time points (Fig. 2*D*). We collected data from five larvae, resulting in a total of 156 internal edges that grow over the course of 24 h. Comparing the initial and final lengths of these edges, we find that edges increase their length by a factor of 1.68±0.05 (Fig. 2*E*). Thus, over the course of 24 h an internal branch on average had increased in length by 68%. Further analysis reveals this increase is independent of the edge orientation and distance from the cell nucleus (*SI Appendix*, Fig. 3 *D* and *E*). Thus, terminal cells grow with a uniform isotropic stretch factor, akin to a constant background inflation.

In summary, we have identified three ways that terminal cell branches can change in time: extension, budding, and internal growth (Fig. 2*F*). We now ask whether the signature of these growth modes can be identified by tracking the global network properties instead of by observing individual events.

### Scaling Laws for Asymptotic Growth Regimes.

We describe the dynamics of network growth across larval development by studying the scaling behavior between pairs of network quantities, which allows for time to be removed from the equation. We consider several statistical quantities of networks, namely: the total sum of all branch lengths L, the convex hull area of the network A, and the average void radius $R_{v}$. The total length L is a measure for the cost of building and maintaining the network, an important value in network optimization problems. Previous work has studied how networks balance the competing requirements to be both space-filling and also self-avoiding (40). The convex hull area A is the two-dimensional analogue of the service volume, which is related to the network demand, or the rate at which material is delivered to the surrounding tissue. The void radius, also known as the distance transform in image analysis applications, is defined for every point in the convex hull by the closest distance from the point to a network edge. $R_{v}$ is calculated by averaging the void radius over all points in the convex hull area and is a measure of the branch density (*SI Appendix*). The relation $L∼A^{\alpha }$ informs how the network fills space as it grows, and $R_{v}∼A^{\beta }$ is a measure of how the empty space between network edges changes over time. Thus, the quantities α and β are geometric measures, containing information on how network edges are packed in space.

We introduce simple examples of growing networks to understand the range of possible scaling exponents. First, we consider a simple square lattice of N×N cells each of side length s. The size of the grid can be increased in two special different ways: by either increasing the size or the number of unit cells. The first mode is a purely scaling mode, where N is constant and s increases (Fig. 3*A*), and the second mode is a purely branching mode, where the N increases and s remains constant (Fig. 3*B*). For these two modes, the values of the scaling exponents can be written explicitly: α=0.5 and β=0.5 for the scaling mode and α≈1.0 and β=0 for the branching mode (see *SI Appendix* for a derivation) (Fig. 3 *D* and *E*). We observe that α=0.5 is the minimal possible scaling exponent if the network remains a single connected component. Furthermore, α=1 indicates that the network maintains a constant density throughout its growth stages. α<1 means that the network becomes less dense and α>1 means that the network density increases over time. Likewise, β=0 indicates that the network gap size is constant over time, β>0 indicates that the average void radius grows, and β<0 indicates that the gaps shrink.

The square grids represent extreme modes of growth. An intermediate example of a network that increases in size through a combination of scaling and branching is a growing Cayley tree, where the edges lengthen and bifurcate in tandem (Fig. 3*C*). We consider a growing tree initialized as a Y-shaped graph that grows into a structure with N generations of edges. Each generation is described by a growth rate 0<k, branching angle 0≤θ≤π, and time of appearance $0<t_{n}$. An edge has length zero before its time of appearance, and length $L_{n}=k\left(t-t_{n}\right)$ for all times $t\geq t_{n}$. We consider an example with N=6, k=1, θ=π/3, and $t_{n}=10\cdot \left(n-1\right)$. For this Cayley tree, the scaling exponents are found to be α=0.84±0.01 and β=0.19±0.003, an intermediate regime between the two extreme cases. In general, calculating α and β over a range of parameters $k,\theta ,t_{n}$ gives a range of scaling laws (*SI Appendix*, Fig. 5). While the Cayley tree is a useful model with slightly more complexity than the growing grids, it lacks the stochasticity observed in the terminal cells.

### Terminal Cells Display an Intermediate Scaling Regime.

For each terminal cell network, the total length, two-dimensional convex hull area, and average void radius were computed. The scaling relations $L∼A^{\alpha }$ and $R_{v}∼A^{\beta }$ were determined using ordinary least-squares regression on a log–log plot, and the results did not change when using major axis regression, a common choice for allometric studies (41). The data span a reasonable dynamic range, with 2.5 orders of magnitude in A and 1.5 orders of magnitude in L. $R_{v}$ displays the smallest range of one order of magnitude. The calculated exponents are α=0.81±0.01 and β=0.24±0.01 (Fig. 3 *F* and *G*). These relations show that terminal cells likely follow a single mode of growth across all larval developmental stages. The scaling laws are an emergent property of the cellular growth dynamics that govern the cell morphology. Notably, the values of α and β indicate that terminal cell growth is neither in the purely scaling nor in the purely branching regime, but displays a combination of the two pure growth modes.

### A Generative Model Decouples the Effects of Branching and Internal Growth.

As internal and branching growth modes display quantitatively distinct scaling behaviors, we devise a model of a growing network that incorporates both dynamic processes. The generative model stochastically adds edge segments to an existing network, similarly to branched self-avoiding random walk models used to study network systems such as the mammary, salivary, and lymphatic glands (2–4).

**Fig. 4.**
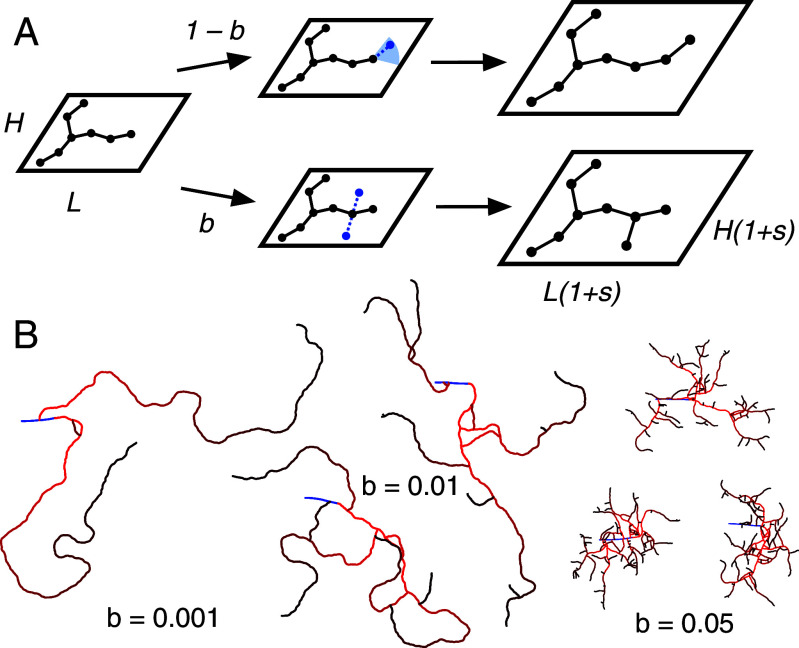
A growing network model with tunable branching and stretching. (*A*) At each time step of the generative algorithm, a new edge is added to the existing network, either by branch extension or by branch budding. Then all network node coordinates undergo a uniform geometric scaling. (*B*) Changing the branching parameter b effectively changes the number of expected branches in the final network. All networks here have N=1,000 and s=0.0001 and are initialized as a single straight segment (shown in blue), but a low b corresponds to a regime with few long branches and a high b corresponds to a dense mesh-like network.

The network is represented as a graph: a collection of nodes with spatial coordinates connected by edges. Each step of the growth algorithm consists of adding an edge of length 1 to the existing network, either at a branch tip (extension), or off the side of an existing branch (budding), followed by a global scaling of the entire network (Fig. 4*A*). Two dimensionless parameters govern the network simulations: the branching factor b and the scaling factor s. The branching factor controls the likelihood of forming a new branch. At each time step a dock node is selected with probability 1−b from the set of existing degree one nodes and with probability b from the set of degree two nodes. Thus, b controls how the number of branches increases over time: For b=0, no new branches are added and existing branches will be extended until they collide with the existing network, and for b=1 the bias toward tip extension is removed and the network will effectively form a tight bundle (Fig. 4*B*). The network geometry depends on the angle each new edge makes with the existing edge. For the case of branch extension, the angle of the new edge is chosen uniformly at random from the range $\left[-\theta_{1},\theta_{1}\right]$. For the case of branch budding, the angle of the new edge is either $\theta_{2}$ or $-\theta_{2}$. For the main simulation results, we set $\theta_{1}=\pi /9$ and $\theta_{2}=\pi /2$, as determined from the branching angle statistics of the terminal cell data, although growth behavior is fairly independent of the specific angle choices (*SI Appendix*, Figs. 2 *F* and *G* and 4). If the new edge causes an overlap, this edge is discarded and the protocol repeats by choosing a new dock node. Once a new edge is added, the network undergoes a uniform background inflation, implemented by multiplying the (x,y) coordinates of each node by 1+s (Fig. 4*A*). This results in exponential growth of the total network length in time.

For an approximation of an early stage right-side terminal cell, we set the initial condition to be a horizontal chain of 30 edges, where new edges are forbidden from being added to the leftmost node in the line. An alternative initial condition is a Y-shaped network, consisting of three chains of 30 edges joined at a single triple vertex. In both cases, the final result is a tree network where nodes have at most three adjacent edges. The initial condition of the network has a slight but negligible effect on the resulting scaling relations.

**Fig. 5.**
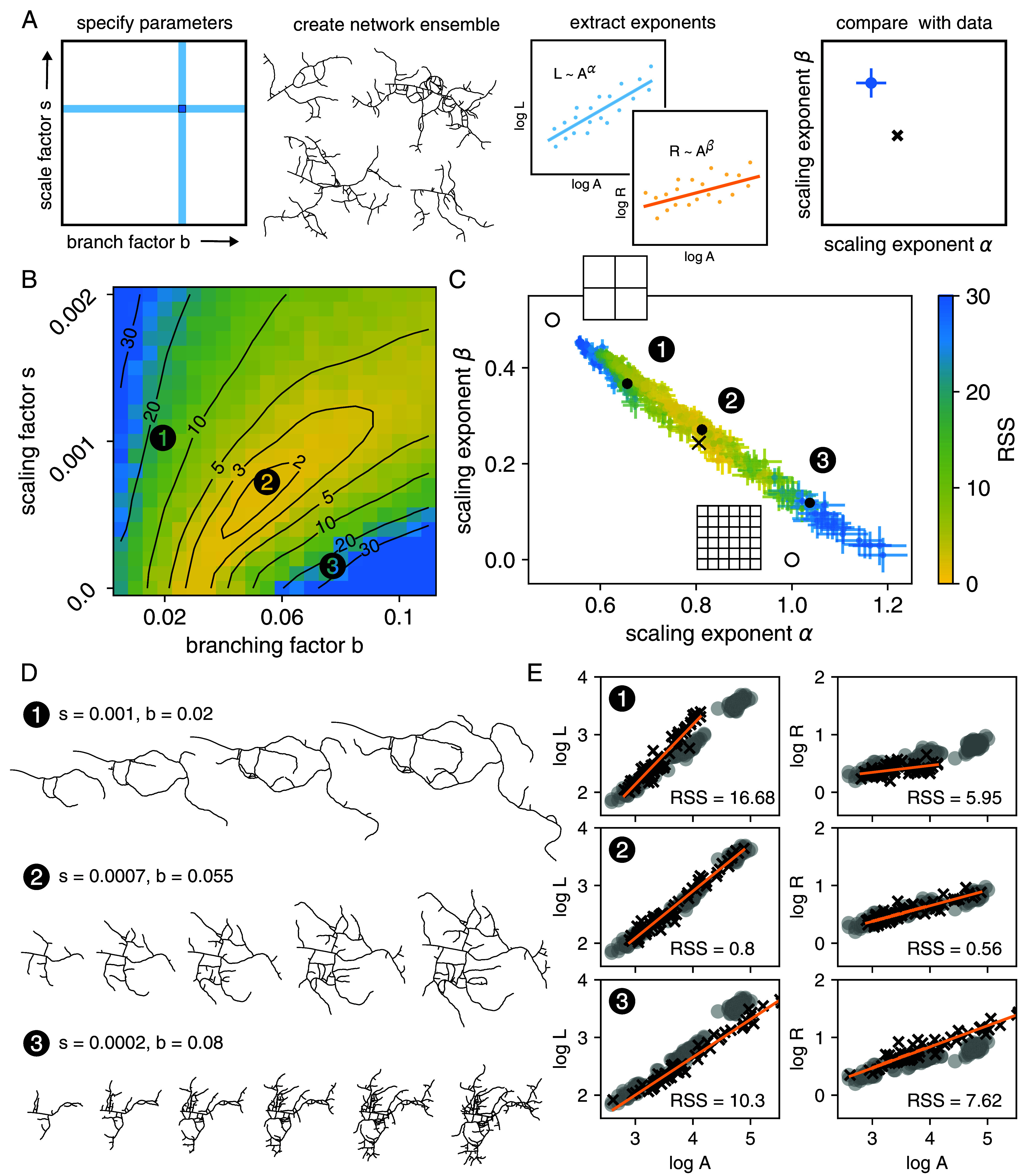
Model fitting and parameter selection. (*A*) Schematic of the model fitting process. Ensembles of simulated networks are generated by sweeping over the parameter space of the scaling factor s (the constant scaling applied at every simulation step) and the branching factor b (the probability of sprouting a new branch at a simulation step). Every value of (s,b) results in a pair (α,β) of extracted scaling exponents. (*B*) Parameter space is colored by the residual sum of squares (RSS) between the best linear fits to the simulations and corresponding terminal cell data. The space has a global minimum with best-fit parameter values (0.0007, 0.055). Three sample parameter sets are indicated: one with high stretching (1), the best-fit set (2), and one with high branching (3). (*C*) Plotting the scaling exponents (α, β) shows a linear relation between the two values. Several reference points are indicated: the purely scaling and purely branching growth modes (open circles), the terminal cell data (×, with the error bars smaller than the marker size), and the three example parameter sets (filled circles). (*D*) One simulated growing network from each of the three sample parameter sets. The number of simulation steps for each network are N= 200, 400, 600, and 800 for (1), N= 200, 400, 600, 800, and 1,000 for (2), and N= 200, 400, 600, 800, 1,000, and 1,200 for (3). (*E*) Examples of the fitting analysis for the sample parameter sets. The ensemble of simulated networks generated from (s, b) (black ×s) is linearly fit (orange), and the goodness of fit is measured by the RSS between the line and the terminal cell data (gray circles).

Varying the stretching and branching parameters changes the growth behavior of the network model, and we calculate the best-fit values explicitly by fitting to the scaling exponents. The exponents are determined by generating an ensemble of networks with fixed parameters s and b but different final sizes N, varying from N=100 to N=2,500. The scaling laws $L∼A^{\alpha }$ and $R_{v}∼A^{\beta }$ are calculated with a linear fit to the network ensemble. Thus, every pair of parameters (s,b) is mapped to a pair of exponents (α,β) (Fig. 5*A*). We find a linear relation between the pairs (α,β) across the sweep of parameter values, indicating an underlying relation between the values L,A, and $R_{v}$ (Fig. 5*C*). Interestingly, this correlation is not seen for a similar analysis of non-self-intersecting Cayley trees, indicating that it is perhaps a geometric consequence of the generative network algorithm. The result of the parameter sweep is compared to the empirical values of the terminal cells $\left(\alpha^{*},\beta^{*}\right)=\left(0.81,0.24\right)$. Goodness of fit is determined by calculating the residual sum of squares (RSS) between the linear fit to the simulation networks and the terminal cell data shown in Fig. 3 *F* and *G*. Points with smaller RSS lie closer to $\left(\alpha^{*},\beta^{*}\right)$, as expected. This method is a reasonable way to test the overall ability of the generative model to fit the observed data values.

Further context is provided by the scaling exponents for the purely scaling and purely branching square grid growth modes. As expected, increasing the scaling factor s yields simulations that approach the purely scaling limit (0.5,0.5). However, the simulation curve does not intersect the point (1,0), the purely branching limit, indicating that it is difficult to achieve a uniform density through a purely stochastic process.

The best-fitting model parameters are recovered using the RSS. The residuals of all points in the two-dimensional parameter space are shown in Fig. 5*B*, and we find a region of best-fitting parameter values, with a minimum around s=0.0007 and b=0.055. This minimum corresponds to networks that grow with analogous developmental scaling behavior to the terminal cells. Examples of generated networks from different regions of parameter space are shown to display the variation in network appearance possible in the model (Fig. 5*D*). The middle row indicates a network that is a good fit to the terminal cell data, and the other two rows give examples of poor fits. Thus, our simple generative algorithm is able to sample a wide space of quantitative network dynamics, and in particular, can recapitulate terminal cell behavior.

We can now connect the optimal parameters of the algorithm (s=0.0007,b=0.055) to the first-order rate constant for stretching of internal edges and the constant rate of the appearance of new branches. The differential form for the exponential stretching of internal branches is given by $dl\left(t\right)/dt=r_{s}l\left(t\right)$, where l(t) is the current length and $r_{s}$ is the first-order constant. We estimated $r_{s}$ using a least squares fit to the time-lapse data, finding $r_{s}=0.022\;\;{\text{h}}^{-1}$. The stretching factor s is the fold increase of the edge length after a small time step dt:l(t+dt)=(1+s)l(t), which in turn can be expressed as $l\left(t+dt\right)=\left(1+r_{s}dt\right)l\left(t\right)$. Thus, $s=r_{s}dt$, and one step of the algorithm corresponds to $s/r_{s}$ hours. Since b is the rate of branching in one step of the algorithm and each branching event adds two branches, the branching rate per unit time is given by $r_{b}=2b/dt=2\cdot b\cdot r_{s}/s$. Combining the experimentally determined value of $r_{s}$ with the optimal model parameters, we find that $r_{b}\approx 3.5$ branches per hour. This value is within a factor of two of a linear fit for the branching rate estimated from a linear fit to cross-sectional data (≈1.7 branches). Thus, based on the combination of time-lapse imaging and model-based fitting of cross-sectional data, we determined that the terminal cells grow through a combination of uniform internal stretching with a doubling time of ≈31.5 h and that new branches are added at a rate of ≈3.5 branches per hour.

## Discussion

Tracheal terminal cell development is a window into distribution network morphogenesis: Over the course of several days, one cell undergoes dramatic morphological changes, growing from a single primary stalk with a few secondary offshoots into a complex structure with around one hundred branches. These cells have a large dynamic range and are thus a prime candidate for studying the general principles of how a supply network adapts to fill a growing territory. We model the terminal cell architecture as a growing network governed by the addition of new branches and the scaling of existing branches. We find that the scaling relations observed in the terminal cells can be reproduced by simulations with fixed values for the branching and scaling parameters throughout the growth process, and identify parameter values that yield the observed growth dynamics. Note that this was not a foregone conclusion: If the terminal cell growth dynamics are variable over time, for instance, if the rate of branching decreases as the cell matures, then the model would not yield a convincing fit. The finding that the observed growth dynamics are consistent with a small set of growth rules, which has been shown to hold as well for complicated multicellular networks, indicates that the terminal cells are a tractable and informative model system for processes that are more difficult to study in vivo, such as organ and vascular formation (42).

The current model is a simplified view of terminal cell growth, ignoring microscopic processes such as vesicle trafficking and lumen formation, information on the limits of the speed of branch growth, and the structure of the underlying muscle that is being supplied, which is not a homogeneous sheet but a multicomponent structure with gaps and layers. In the model, the angle of the budding branch is chosen from a simple distribution based on the orientation of the parent branch. However, a distinct phase of new branch formation is the filopodial protrusion, consisting of several wispy extensions, which then matures into the full branch. The process of how the filopodium selects which extensions are pruned and which are reinforced is not well understood but likely involves an environmental sensing component. The most realistic modeling approach for the growing trachea involves simulating the hypoxia-induced production of ligand in the muscle and inducing a growth response to regions where ligand binds to the trachea membrane receptors. Such a model requires information on the oxygen gradient field in the surrounding tissue, the dynamics of ligand production, and the response of branch extension and budding to receptor activation. In summary, the simple model presented in this work can certainly be improved by including more sophisticated mechanisms of environmental feedback into the growth decisions. Until these mechanisms have been more thoroughly understood, they remain an area of speculation.

In particular, the mechanism through which network scaling occurs is an open question. We have shown that terminal cell branches are not immutable, but continue to extend throughout development. One possible explanation is that material is constantly being added to the bulk of the branches through membrane trafficking. This type of mechanism has been proposed for *Drosophila* dendritic arborization neurons, but an analogue for trachea has not been found (43). The mechanism for bud formation requires more attention as well. In the generative model, the low values for the branching parameter are motivated by the biological intuition that forming a new filopodia in the middle of a branch is a more energy-intensive process than building on the existing filopodia that exist at all branch tips. Testing this theory requires a deeper understanding of how a filopodial complex forms. These open questions can likely be clarified by continuously tracking tracheal development in a single larva over multiple days.

Our work solidifies the utility of examining scaling laws to understand the underlying conserved features of a network ensemble, resulting in a compact description of complex dynamic structures. While we consider only a single type of growth algorithm in this paper, as mentioned before there are many ways to alter this model, for instance by introducing a lateral inhibition effect that suppresses bud formation around existing branches, or a branching parameter that is dependent on network features such as the age of the budding location or the local density of neighboring branches. These additional rules would result in better overall model fits, as a general rule of additional model features, and comparing the sweep of simulation results to the observed data scaling exponents is a reliable way to quantify the improvement of feature expansion. Because these methods provide a straightforward way to characterize network structure, they can be utilized in future studies of mutations affecting terminal cells. For instance, an increased value of α indicates that the mutant networks become more dense than the wild-type networks over time. This effect could arise from genetic perturbations that interfere with the chemical sensing capabilities of the terminal cells, or from environmental perturbations such as rearing animals in hypoxic environments, which is known to result in increased terminal cell branching (25, 44, 45).

The interpretation of scaling laws in networks is context-dependent. For instance, river networks have been shown to follow scaling laws, which are related to their self-similarity properties (28, 46, 47). Another example is dendritic trees: A tight relation between the total length and the number of nodes exhibited by neurons has been shown to hold across many cell types and species, suggesting that this level of connectivity is a result of the neuron optimizing its wiring for signal propagation (48, 49). The scaling exponent for dendritic arborization neurons, which tile the surface of the larval body, has been determined to be α=0.78, which is close to the value that we find for terminal cells α=0.81 (50). The similarity of these two scaling exponents is unexpected, since these neurons follow completely different developmental dynamics, establishing their structure through the exploration of space by actively protruding and retracting tips (51–53). This may suggest that these laws may be a consequence of how the larval body size increases over time.

## Materials and Methods

### Fly Strains.

Zasp:GFP flies were generously provided by Mary Baylies. All embryos and larvae imaged and traced (see below) for this study were the progeny of female btl > gal4, uas > gfp flies crossed to male UAS > mCherry RNAi (BL #35785) flies.

### Whole Mount Larval Imaging.

Embryos were collected for three hours on apple juice agar plates with yeast paste and maintained at 25 °C. Larvae of the desired instar were placed in a drop of glycerol on a glass slide and heat-fixed on a 70 °C heat block (1 s for first instar, 3 s for second instar, 7 s for third instar). Heat-fixed larvae were immediately imaged on a Nikon C2 confocal microscope. Larval instar was determined by the time after egg lay and by the morphology of the anterior spiracles. Fluorescent imaging was performed on a point scanning confocal microscope with a 20× objective using the 488 nm laser. *Z*-stacks were taken with a step size of 0.8 μm.

### Larval Time-Lapse Imaging.

Embryos were collected for three hours at 25 °C on apple juice agar plates containing yeast paste. First instar larvae were collected 24 h later and were imaged on a Nikon C2 confocal microscope. To prevent movement during imaging, larvae were adhered to double-sided tape on a glass slide and positioned dorsal side up to allow visualization of the dorsal terminal cells. A coverslip was laid over the top of the larvae, with aluminum foil spacers placed beside the larvae to prevent them from being crushed. Immediately after imaging, first instars were placed back onto apple juice agar plates containing yeast paste and maintained at 25 °C. Larvae were imaged at regular intervals (following the same procedure) until the late L3 stage. Larval instar was determined by the time after egg lay and by the morphology of the anterior spiracles.

### Fixation and Immunofluorescence.

Homozygous btl > gal4, uas > gfp were dissected, fixed, and stained (33) with chick anti-GFP (1:1000, Thermoscientific, #A10262) and Alexa Fluor 488 goat anti-chicken IgG (H+L) (1:1000, Thermoscientific, #A11039) to observe filopodia in Fig. 2*A*.

### Image Processing.

Micrographs were processed using the trace tool of Fiji plugin SNT, and trace information was exported in a TRACES file (54, 55). In this format, each terminal cell branch is discretized into nodes placed automatically, on average a distance of 0.5 μm apart. Trace information was converted into a graph object and analyzed using the python NetworkX package (56). The fill function of SNT was used to make the terminal cell structures in *SI Appendix*, Fig. 1.

## Supplementary Material

Appendix 01 (PDF)

## Data Availability

Segmented network data, network analysis code, and simulation code data have been deposited in GitHub (https://github.com/tgavrilchenko/terminal-cells) (57).

## References

[1] R. J. Metzger, O. D. Klein, G. R. Martin, M. A. Krasnow, The branching programme of mouse lung development. Nature **453**, 745–750 (2008). 18463632 10.1038/nature07005 PMC2892995

[2] E. Hannezo , A unifying theory of branching morphogenesis. Cell **171**, 242–255 (2017). 28938116 10.1016/j.cell.2017.08.026 PMC5610190

[3] I. Bordeu, L. Chatzeli, B. D. Simons, Inflationary theory of branching morphogenesis in the mouse salivary gland. Nat. Commun. **14**, 3422 (2023). 37296120 10.1038/s41467-023-39124-x PMC10256724

[4] M. C. Uçar , Self-organized and directed branching results in optimal coverage in developing dermal lymphatic networks. Nat. Commun. **14**, 5878 (2023). 37735168 10.1038/s41467-023-41456-7 PMC10514270

[5] Y. Coudert, S. Harris, B. Charrier, Design principles of branching morphogenesis in filamentous organisms. Curr. Biol. **29**, R1149–R1162 (2019). 31689405 10.1016/j.cub.2019.09.021

[6] M. C. Uçar , Theory of branching morphogenesis by local interactions and global guidance. Nat. Commun. **12**, 1–10 (2021). 34819507 10.1038/s41467-021-27135-5 PMC8613190

[7] A. Runions *et al*., “Modeling and visualization of leaf venation patterns” in *ACM SIGGRAPH 2005 Papers, SIGGRAPH ’05*, M. Gross, Ed. (Association for Computing Machinery, New York, NY, 2005), pp. 702–711.

[8] A. Ruiz-Sobrino , Space colonization by branching trachea explains the morphospace of a simple respiratory organ. Dev. Biol. **462**, 50–59 (2020). 32109442 10.1016/j.ydbio.2020.02.005

[9] J. A. Davies, P. Hohenstein, C. Chang, R. Berry, A self-avoidance mechanism in patterning of the urinary collecting duct tree. BMC Dev. Biol. **14**, 1–12 (2014). 10.1186/s12861-014-0035-8 PMC4448276 25205115

[10] V. Wigglesworth, W. Lee, The supply of oxygen to the flight muscles of insects: A theory of tracheole physiology. Tissue Cell **14**, 501–518 (1982). 7147227 10.1016/0040-8166(82)90043-x

[11] A. Schmitz, S. F. Perry, Stereological determination of tracheal volume and diffusing capacity of the tracheal walls in the stick insect carausius morosus (phasmatodea, lonchodidae). Physiol. Biochem. Zool. **72**, 205–218 (1999). 10068624 10.1086/316655

[12] C. Cabernard, M. Neumann, M. Affolter, Cellular and molecular mechanisms involved in branching morphogenesis of the *Drosophila* tracheal system. J. Appl. Physiol. **97**, 2347–2353 (2004). 15531575 10.1152/japplphysiol.00435.2004

[13] L. Centanin, T. A. Gorr, P. Wappner, Tracheal remodelling in response to hypoxia. J. Insect Physiol. **56**, 447–454 (2010). 19482033 10.1016/j.jinsphys.2009.05.008 PMC2862287

[14] A. Ochoa-Espinosa, M. Affolter, Branching morphogenesis: From cells to organs and back. Cold Spring Harb. Perspect. Biol. **4**, a008243 (2012). 22798543 10.1101/cshperspect.a008243 PMC3475165

[15] E. Hannezo, B. D. Simons, Multiscale dynamics of branching morphogenesis. Curr. Opin. Cell Biol. **60**, 99–105 (2019). 31181348 10.1016/j.ceb.2019.04.008

[16] T. Weis-Fogh, Diffusion in insect wing muscle, the most active tissue known. J. Exp. Biol. **41**, 229–256 (1964). 14187297 10.1242/jeb.41.2.229

[17] J. Sauerwald, W. Backer, T. Matzat, F. Schnorrer, S. Luschnig, Matrix metalloproteinase 1 modulates invasive behavior of tracheal branches during entry into *Drosophila* flight muscles. eLife **8**, e48857 (2019). 31577228 10.7554/eLife.48857 PMC6795481

[18] A. S. Ghabrial, S. Luschnig, M. M. Metzstein, M. A. Krasnow, Branching morphogenesis of the *Drosophila* tracheal system. Annu. Rev. Cell Dev. Biol. **19**, 623–647 (2003). 14570584 10.1146/annurev.cellbio.19.031403.160043

[19] K. Kato, T. Chihara, S. Hayashi, Hedgehog and decapentaplegic instruct polarized growth of cell extensions in the *Drosophila* trachea. Development **131**, 5253–5261 (2004). 15456724 10.1242/dev.01404

[20] M. J. Klowden, *Respiratory Systems* (Academic Press, San Diego, CA, ed. 3, 2013), pp. 445–474.

[21] C. Samakovlis , Development of the *Drosophila* tracheal system occurs by a series of morphologically distinct but genetically coupled branching events. Development **122**, 1395–1407 (1996). 8625828 10.1242/dev.122.5.1395

[22] N. Hacohen, S. Kramer, D. Sutherland, Y. Hiromi, M. A. Krasnow, sprouty encodes a novel antagonist of FGF signaling that patterns apical branching of the *Drosophila* airways. Cell **92**, 253–263 (1998). 9458049 10.1016/s0092-8674(00)80919-8

[23] S. Hayashi, T. Kondo, Development and function of the *Drosophila* tracheal system. Genetics **209**, 367–380 (2018). 29844090 10.1534/genetics.117.300167 PMC5972413

[24] B. T. Best, Single-cell branching morphogenesis in the *Drosophila* trachea. Dev. Biol. **451**, 5–15 (2019). 30529233 10.1016/j.ydbio.2018.12.001

[25] J. Jarecki, E. Johnson, M. A. Krasnow, Oxygen regulation of airway branching in *Drosophila* is mediated by branchless FGF. Cell **99**, 211–220 (1999). 10535739 10.1016/s0092-8674(00)81652-9

[26] T. A. Jones, M. M. Metzstein, A novel function for the par complex in subcellular morphogenesis of tracheal terminal cells in *Drosophila* melanogaster. Genetics **189**, 153–164 (2011). 21750259 10.1534/genetics.111.130351 PMC3176136

[27] H. Cuntz, F. Forstner, A. Borst, M. Häusser, One rule to grow them all: A general theory of neuronal branching and its practical application. PLoS Comput. Biol. **6**, e1000877 (2010). 20700495 10.1371/journal.pcbi.1000877 PMC2916857

[28] A. Maritan, A. Rinaldo, R. Rigon, A. Giacometti, I. Rodríguez-Iturbe, Scaling laws for river networks. Phys. Rev. E **53**, 1510 (1996). 10.1103/physreve.53.1510 9964414

[29] J. R. Banavar, A. Maritan, A. Rinaldo, Size and form in efficient transportation networks. Nature **399**, 130–132 (1999). 10335841 10.1038/20144

[30] M. M. Baer, A. Bilstein, M. Leptin, A clonal genetic screen for mutants causing defects in larval tracheal morphogenesis in *Drosophila*. Genetics **176**, 2279–2291 (2007). 17603107 10.1534/genetics.107.074088 PMC1950631

[31] A. S. Ghabrial, B. P. Levi, M. A. Krasnow, A systematic screen for tube morphogenesis and branching genes in the *Drosophila* tracheal system. PLoS Genet. **7**, e1002087 (2011). 21750678 10.1371/journal.pgen.1002087 PMC3131284

[32] D. Ricolo, J. Castro-Ribera, S. J. Araújo, Cytoskeletal players in single-cell branching morphogenesis. Dev. Biol. **477**, 22–34 (2021). 34004181 10.1016/j.ydbio.2021.05.001

[33] C. M. Bourne, D. C. Lai, J. Schottenfeld-Roames, Regulators of the secretory pathway have distinct inputs into single-celled branching morphogenesis and seamless tube formation in the *Drosophila* trachea. Dev. Biol. **490**, 100–109 (2022). 35870495 10.1016/j.ydbio.2022.07.005

[34] R. Pradhan, V. Urbieta-Ortiz, S. Kumar, R. Mathew, L. Ríos-Barrera, Shaping subcellular tubes through vesicle trafficking: Common and distinct pathways. Semin. Cell Dev. Biol. **133**, 74–82 (2023). 35365398 10.1016/j.semcdb.2022.03.024

[35] L. Gervais, J. Casanova, In vivo coupling of cell elongation and lumen formation in a single cell. Curr. Biol. **20**, 359–366 (2010). 20137948 10.1016/j.cub.2009.12.043

[36] J. Schottenfeld-Roames, A. S. Ghabrial, Whacked and Rab35 polarize dynein-motor-complex-dependent seamless tube growth. Nat. Cell Biol. **14**, 386–393 (2012). 22407366 10.1038/ncb2454 PMC3334817

[37] N. JayaNandanan, R. Mathew, M. Leptin, Guidance of subcellular tubulogenesis by actin under the control of a synaptotagmin-like protein and moesin. Nat. Commun. **5**, 3036 (2014). 24413568 10.1038/ncomms4036 PMC3945880

[38] F. De Smet, I. Segura, K. De Bock, P. J. Hohensinner, P. Carmeliet, Mechanisms of vessel branching: Filopodia on endothelial tip cells lead the way. Arterioscler. Thromb. Vasc. Biol. **29**, 639–649 (2009). 19265031 10.1161/ATVBAHA.109.185165

[39] T. A. Jones, L. S. Nikolova, A. Schjelderup, M. M. Metzstein, Exocyst-mediated membrane trafficking is required for branch outgrowth in *Drosophila* tracheal terminal cells. Dev. Biol. **390**, 41–50 (2014). 24607370 10.1016/j.ydbio.2014.02.021 PMC4041209

[40] D. Hunt, V. M. Savage, Asymmetries arising from the space-filling nature of vascular networks. Phys. Rev. E **93**, 062305 (2016). 27415278 10.1103/PhysRevE.93.062305

[41] A. W. Shingleton, Which line to follow? The utility of different line-fitting methods to capture the mechanism of morphological scaling Integr. Comp. Biol. **59**, 1399–1410 (2019). 31120495 10.1093/icb/icz059

[42] D. J. Andrew, A. J. Ewald, Morphogenesis of epithelial tubes: Insights into tube formation, elongation, and elaboration. Dev. Biol. **341**, 34–55 (2010). 19778532 10.1016/j.ydbio.2009.09.024 PMC2854166

[43] Y. Peng , Regulation of dendrite growth and maintenance by exocytosis. J. Cell Sci. **128**, 4279–4292 (2015). 26483382 10.1242/jcs.174771 PMC4712815

[44] L. Centanin , Cell autonomy of HIF effects in *Drosophila*: Tracheal cells sense hypoxia and induce terminal branch sprouting. Dev. cell **14**, 547–558 (2008). 18410730 10.1016/j.devcel.2008.01.020

[45] J. F. Harrison , Developmental plasticity and stability in the tracheal networks supplying *Drosophila* flight muscle in response to rearing oxygen level. J. Insect Physiol. **106**, 189–198 (2018). 28927826 10.1016/j.jinsphys.2017.09.006

[46] P. S. Dodds, D. H. Rothman, Unified view of scaling laws for river networks. Phys. Rev. E **59**, 4865 (1999). 10.1103/physreve.59.4865 11969437

[47] S. Żukowski, P. Morawiecki, H. Seybold, P. Szymczak, Through history to growth dynamics: Deciphering the evolution of spatial networks. Sci. Rep. **12**, 20407 (2022). 36437299 10.1038/s41598-022-24656-x PMC9701698

[48] H. Cuntz, A. Mathy, M. Häusser, A scaling law derived from optimal dendritic wiring. Proc. Natl. Acad. Sci. U.S.A. **109**, 11014–11018 (2012). 22715290 10.1073/pnas.1200430109 PMC3390826

[49] H. Cuntz , Preserving neural function under extreme scaling. PLoS One **8**, e71540 (2013). 23977069 10.1371/journal.pone.0071540 PMC3747245

[50] L. Baltruschat, G. Tavosanis, H. Cuntz, A developmental stretch-and-fill process that optimises dendritic wiring. bioRxiv [Preprint] (2020). 10.1101/2020.07.07.191064 (Accessed 28 December 2023).

[51] A. Palavalli, N. Tizón-Escamilla, J. F. Rupprecht, T. Lecuit, Deterministic and stochastic rules of branching govern dendrite morphogenesis of sensory neurons. Curr. Biol. **31**, 459–472 (2021). 33212017 10.1016/j.cub.2020.10.054

[52] S. Shree , Dynamic instability of dendrite tips generates the highly branched morphologies of sensory neurons. Sci. Adv. **8**, eabn0080 (2022). 35767611 10.1126/sciadv.abn0080 PMC9242452

[53] M. Liao, A. D. Bird, H. Cuntz, J. Howard, Topology recapitulates morphogenesis of neuronal dendrites. Cell Rep. **42**, 113268 (2023). 38007691 10.1016/j.celrep.2023.113268 PMC10756852

[54] C. Arshadi, U. Günther, M. Eddison, K. I. S. Harrington, T. A. Ferreira, SNT: A unifying toolbox for quantification of neuronal anatomy. Nat. Methods **18**, 374–377 (2021). 33795878 10.1038/s41592-021-01105-7

[55] J. Schindelin , Fiji: An open-source platform for biological-image analysis. Nat. Methods **9**, 676–682 (2012). 22743772 10.1038/nmeth.2019 PMC3855844

[56] A. Hagberg, P. Swart, D. S. Chult, “Exploring network structure, dynamics, and function using networkx” (Tech. Rep. LA-UR-08-05495, Los Alamos National Lab LANL, Los Alamos, NM, 2008).

[57] T. Gavrilchenko, Data and code from “The *Drosophila* tracheal terminal cell as a model for branching morphogenesis.” GitHub. https://github.com/tgavrilchenko/terminal-cells. Deposited 20 May 2024. 10.1073/pnas.2404462121 PMC11474054 39356666

