# Supplementary material for: The Drosophila tracheal terminal cell as a model for branching morphogenesis
Source: Proc Natl Acad Sci U S A. 2024 Oct 2;121(41):e2404462121. doi: 10.1073/pnas.2404462121 (PMC11474054; doi:10.1073/pnas.2404462121)
Supplement: Supplementary file 1 — Appendix 01 (PDF) [file pnas.2404462121.sapp.pdf]

Supporting Information: The *Drosophila* tracheal terminal cell as a model for branching morphogenesis

Tatyana Gavrilchenko,[1] Alison G. Simpkins,[2] Tanner Simpson,[3] Lena A. Barrett,[2,4]
Pauline Hansen,[2] Stanislav Y. Shvartsman,[1,2,3] and Jodi Schottenfeld-Roames[3]

[1] *Flatiron Institute, Simons Foundation, New York, NY 10010*
[2] *Lewis-Sigler Institute for Integrative Genomics, Princeton University, Princeton, NJ 08544*
[3] *Department of Molecular Biology, Princeton University, Princeton, NJ 08544*
[4] *McKinsey & Company, Philadelphia, PA 19104*

I. PROJECTING TERMINAL CELLS INTO 2D

Because the terminal cells cover flat muscle sheets, network geometries are expected to be mostly flat. This is confirmed by measuring the span of the terminal cells in three dimensions and observing that the depth is negligible compared to both the height and width (Supplementary Figure 2B-C). Thus, for analysis terminal cell coordinates are projected into the best-fit two dimensional plane, determined by singular value decomposition. Measuring the difference in length change between the initial 3D networks and the projected 2D networks yielded a small change, indicating that the planar approximation for terminal cells is appropriate (Supplementary Figure 2D).

II. VOID RADIUS CALCULATION

The void radius is used as a measure of density of network branches. To calculate this value for a terminal cell network, the convex hull was computed and a 300×300 grid was superimposed to cover the hull area. For each grid point, the closest Euclidean distance to either a network node or the hull boundary was computed. The final R_v is the mean closest distance over all grid points.

III. ANGLE STATISTICS INFORMING THE GENERATIVE MODEL

The angle of newly-placed tip extension edges in the generative network model is drawn uniformly at random from the interval $[-\theta_1, \theta_1]$. To set the value of θ_1, we extract a measure akin to the persistence length of a terminal cell branch. Branches are discretized into segments of length $s = 5\,\mu\text{m}$, $10\,\mu\text{m}$, or $20\,\mu\text{m}$ for L1, L2, and L3, respectively, and the angle between adjacent segments is computed, with histograms over all segments per instar shown in Supplementary Figure 2F. For the third instar, the distribution mean and standard deviation are $-4^\circ \pm 21^\circ$, so we set $\theta_1 = \pi/9 \approx 20^\circ$. Branching angle statistics are shown in Supplementary Figure 2G. The angle of newly-placed budding edges in the generative network model is set to symmetrically be either θ_2 or $-\theta_2$, with $\theta_2 = \pi/2$.

IV. GROWING CAYLEY TREE MODEL

In principle, $3N$ parameters are needed to describe a three-fold symmetric tree with N generations: k_n, θ_n, and t_n for each of the N generations. To simplify the model, we introduce recursive relations for the branch generations. The growth rate of the first branch k_1 and branching angle of the first split θ_1 are set as two parameters, and the subsequent parameters for all daughter branches are derived recursively from the value of the mother parameters. We let $k_{n+1} = \alpha k_n$ and call α the growth ratio, which controls the growth relation between subsequent generations: $\alpha = 1$ means that all branches grow at the same rate, $\alpha < 1$ means that all daughter branches grow slower than the mother, and $\alpha > 1$ means that all daughter branches grow faster than the mother. Similarly we take $\theta_{n+1} = \beta\theta_n$ and call β the branching angle ratio: $\beta = 1$ means that all branches in the tree will have the same angle, $\beta < 1$ means that the branch angles become narrower with subsequent generations, and $\beta > 1$ means that the branch angles become wider. Finally, we set the generations to appear at times $t_n = rn$, where r is the branching rate. Thus, six parameters are necessary to describe such trees: N, k_1, θ_1, α, β, and r. For the Cayley trees, increasing k corresponds to increasing the stretching rate and increasing t_n increases the branching rate, thus driving the system to one of the extreme cases explained by the two grid examples.

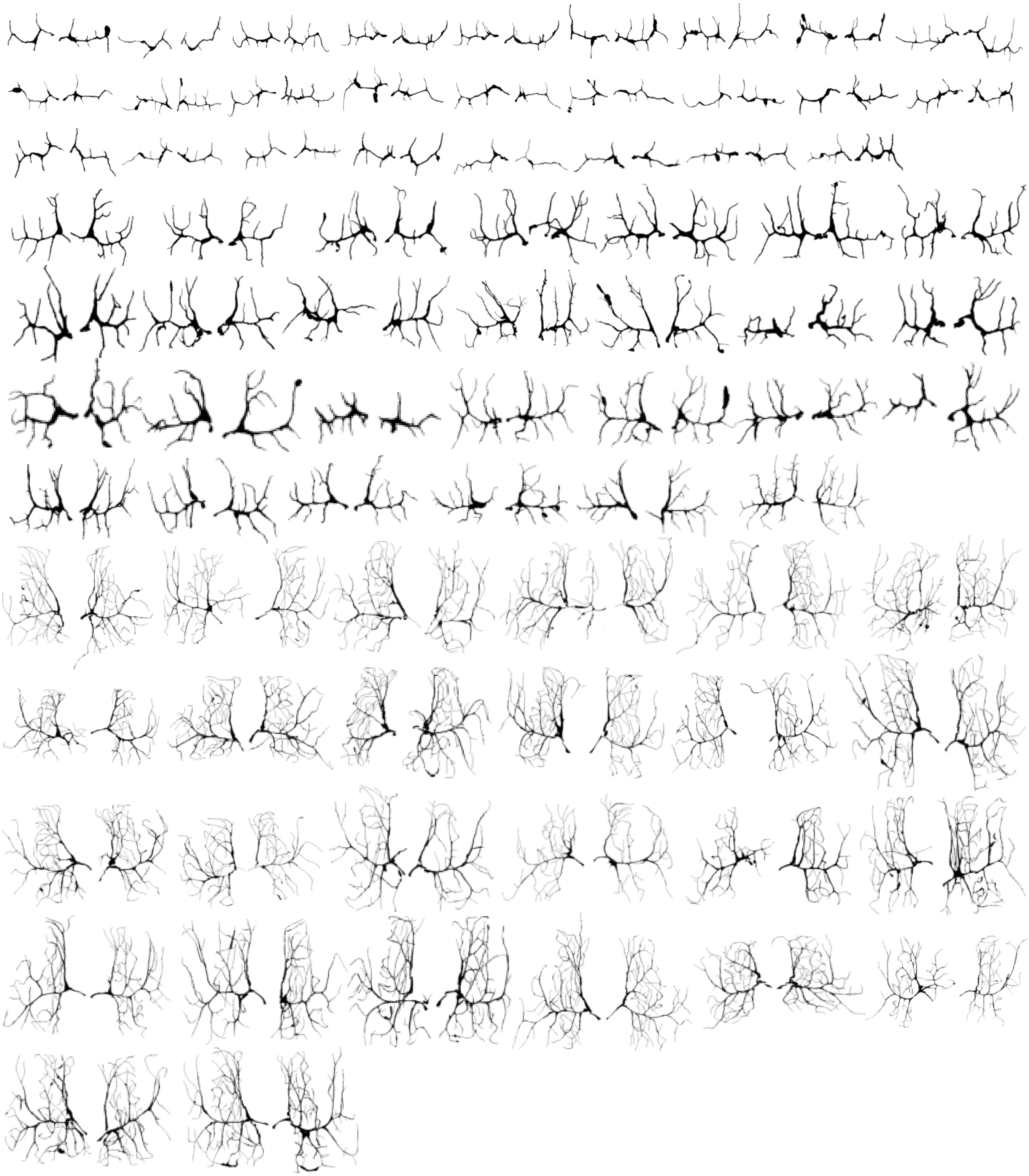

SUPPORTING FIG. 1. The full trachea terminal cell data set, consisting of 52 terminal cells at the L1 stage, 54 at the L2 stage, and 56 at the L3 stage, with cells appearing in left-right pairs. See Figure 1F-F″ for scale information.

V. GROWTH EXPONENTS FOR THE SQUARE GRID

Consider a $N \times N$ square grid, where each square has side length s. The convex hull area of the grid is $A = (Ns)^2$. The number of edges is $N_e = 2N(N+1)$, so the sum of all edge lengths is $L = 2N(N+1)s$. We can use these two equations to find the scaling law $L \sim A^\alpha$ in two special cases of growth. In the case of purely stretching growth, N is constant whereas s increases. Therefore, $A \sim s^2$ and $L \sim s$; therefore, $L \sim A^{0.5}$. In the case of purely self-similar growth, N increases whereas s is constant. Therefore, $L \sim N(N+1)$ and $A \sim N^2$. This gives $L \sim A + A^{0.5}$, so to first order, $L \sim A$.

For a simple grid, we can also write down the explicit form for the average void radius R_v, defined as the mean closest distance from an arbitrary point within the convex hull area of the grid to an edge of the grid. In other words, given a square of side length s, what is the expected distance from a point inside the square to the boundary? We divide the square into eight equal right triangles, each of area $s^2/8$. Considering the triangle in the bottom left corner, the shortest distance to the grid is just the vertical distance from the bottom edge. Thus, the mean void radius is calculated by integrating the vertical distance over the triangle and normalizing by the triangle area: $R_V = \frac{8}{s^2}\int_0^{s/2} dx \int_0^x y\, dy = \frac{s}{6}$ Thus, $R_V \sim s$. So in the case of purely stretching growth, $R_V \sim A^{0.5}$, and in the case of purely self-similar growth, R_v is constant, which makes sense from examining the grids.

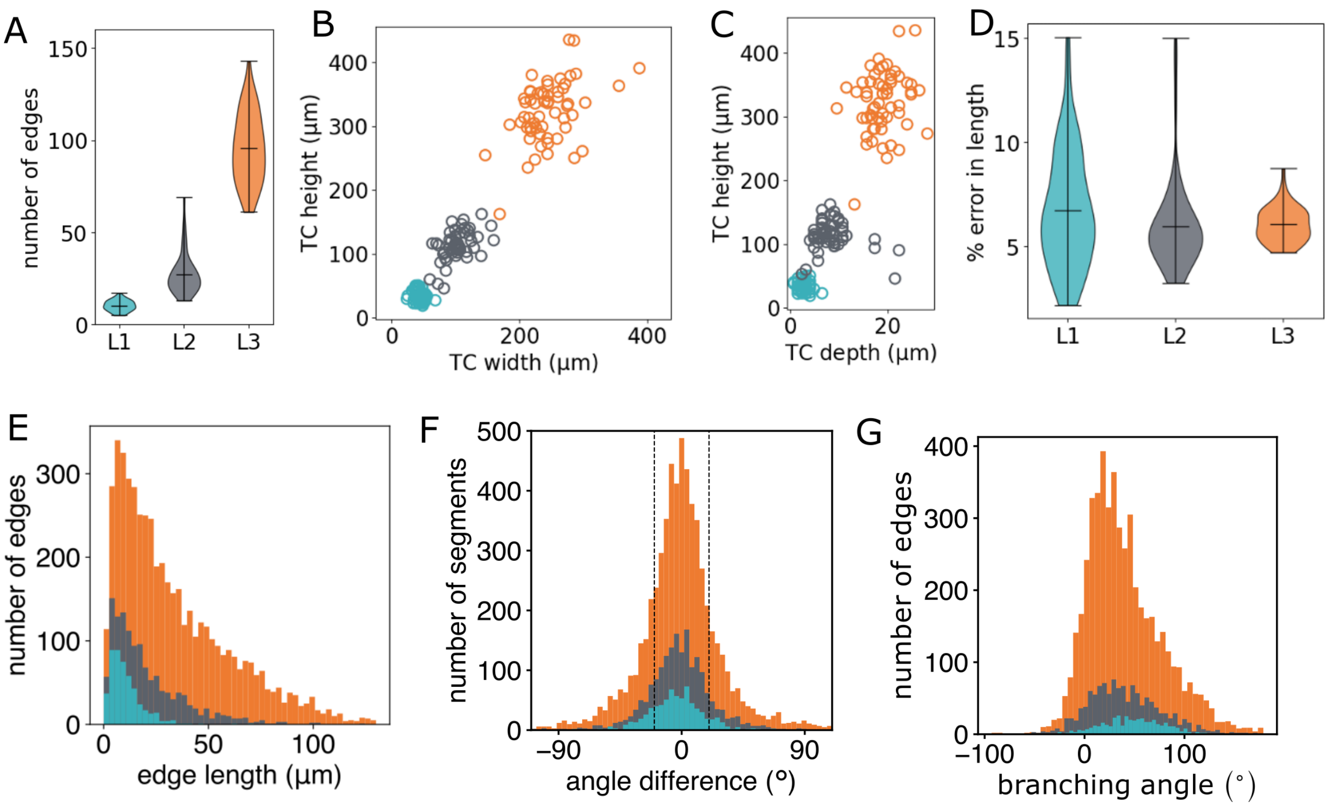

SUPPORTING FIG. 2. (**A**) Statistics for the average number of edges in terminal cells of the three different stages. L1: 10 ± 3, L2: 27 ± 10, L3: 95 ± 22. (**B**) Distributions of terminal cell sizes in terms of the range that a network covers is a given direction. Here, height refers to the range in the apical-posterior dimension and width refers to the range of the lateral dimension. (**C**) Distributions of the terminal cell sizes that show the difference of scale between the height of a terminal cell (y-dimension) and its depth (z-dimension). This shows that the terminal cells are mostly flat, as the mean depth is roughly 5 percent of the mean height. (**D**) For network analysis, three-dimensional terminal cell networks are projected into the best-fit plane to result in two-dimensional networks will a small number of overlapping branches. To confirm that this is a reasonable approximation to make and that this method does not skew network metrics, we measure the total length of the three-dimensional network L_{3D} and the total length of the two-dimensional network L_{2D} and consider the error between the two measurements $error = 100(1 - L_{2D}/L_{3D})$. The closer this error is to zero, the more two-dimensional the network. For the three network stages, the average error and its standard deviation were 6.7 ± 2.8, 6.0 ± 2.2, 6.0 ± 0.8, indicating that terminal cells are approximately flat over all larval stages. (**E**) Edge length distributions over the three larval stages. (**F**) Branching angle distributions over the three larval stages.

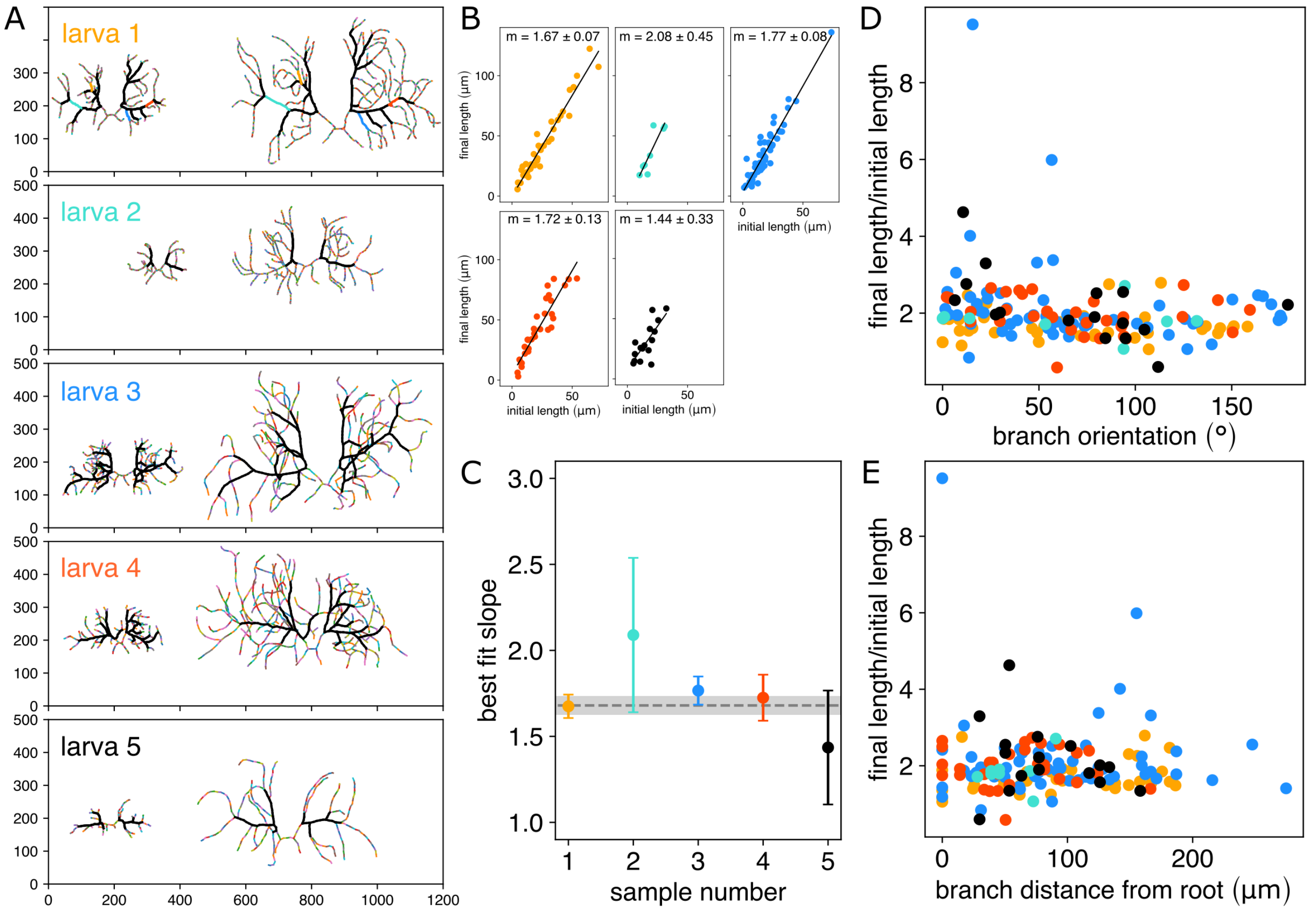

SUPPORTING FIG. 3. **Additional replicates of the 24 hour internal growth experiment.** **(A)** The first sample is the internal growth experiment from Figure 2 of the main text and samples 2-5 are replicates of this time lapse experiment. Initial measurements are taken with late L2 stage larvae and final measurements are made 24 hours later, when the larva are in the early L3 stage. Internal branches present in both stages were identified and their lengths were measured. Samples have different amounts of internal branches depending on the development of the terminal cells. The number of internal branches is 38, 8, 62, 32, and 16, for samples 1-5 respectively. Internal branches are shown in black, and other edge segments are colored. **(B)** Individual linear fits for each of the 5 larva, with extracted slopes and errors indicated. **(C)** The slope and slope error for each of the five samples. All data points lie within the error bars of other points, indicating that all samples exhibit a similar internal growth rate. Internal growth is independent of both the angle of the branch **(D)** and the distance of the branch to the terminal cell nucleus **(E)**. Here, the angle of the branch orientation is measured from the vertical, with left-handed and right-handed angles treated identically, and the branch distance is measured by the Euclidean distance between the closest branch node and the approximate location of the nucleus. Both measurements are made in the L3 stage of the terminal cell. When fit to a line, both data sets give a slope close to 0 (-0.00005 ± 0.00128 and -0.003 ± 0.001, respectively), indicating that the pairs of variables are uncorrelated.

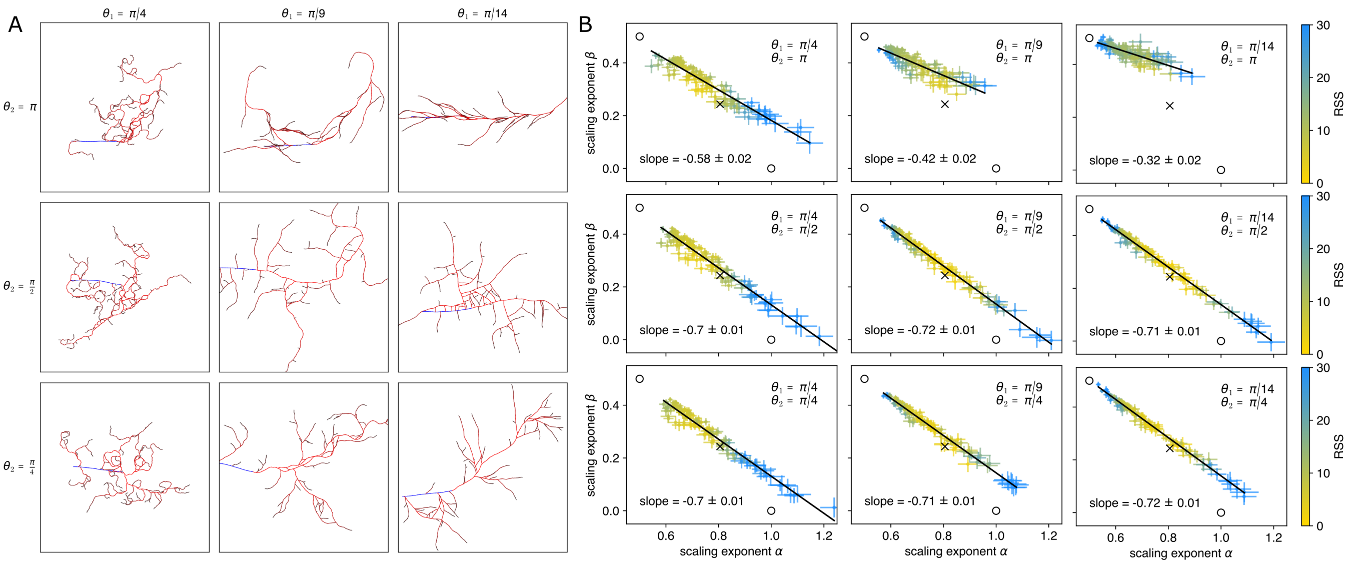

SUPPORTING FIG. 4. **Simulations with alternate angle parameter choices.** **(A)** Sample parameter networks with several alternate parameter choices. The center panel is the parameter set used in the main text. Here θ_1 sets the range of possible angles chosen for tip extension, and a smaller choice of θ_1 effectively increases the persistence length of a network branch. A newly budded branch forms with angle either θ_2 or $-\theta_2$ with the existing branch. **(B)** Corresponding fit exponents for each new set of parameters. These results indicate that the choice of θ_2 has a stronger impact on the outcome of the final scaling relations, and that the simulations are relatively insensitive to the choice of θ_1.

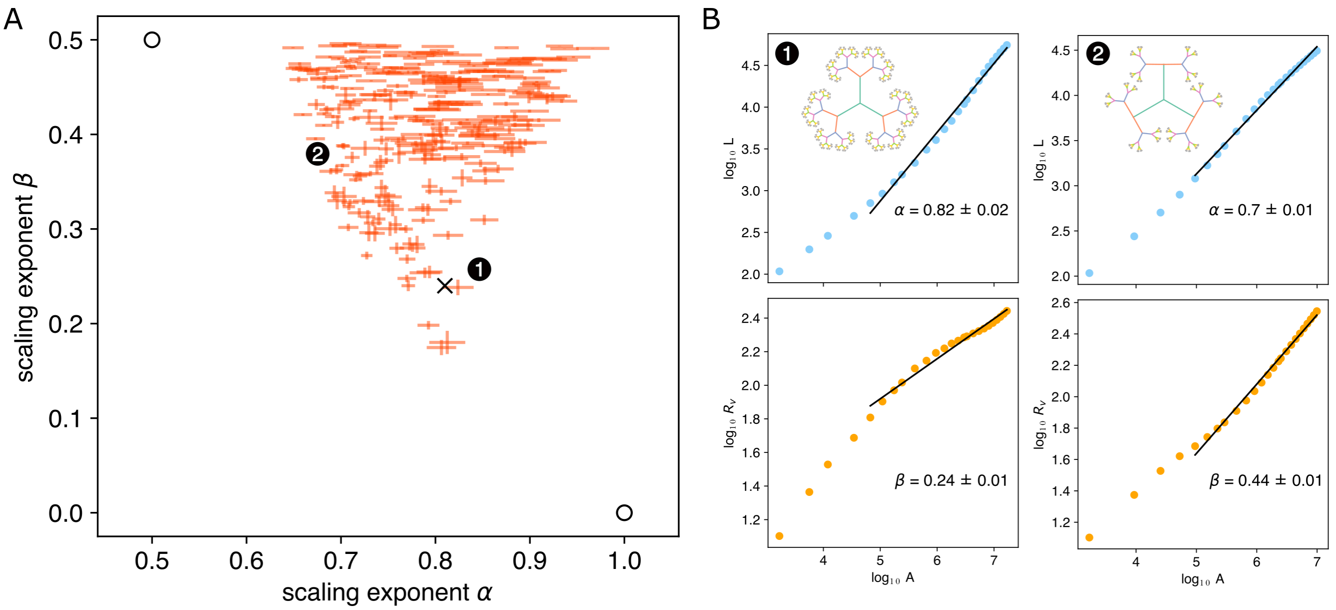

SUPPORTING FIG. 5. **Computing the scaling exponents for a set of Cayley trees.** **(A)** We simulate an ensemble of Cayley trees, with parameter values chosen independently from reasonable parameter ranges. Non-self-intersecting trees are kept and self-intersecting trees are discarded. The scaling exponents are extracted as in Figure 5 of the main text, and the resulting pairs of α and β are shown for different parameter choices. Unlike the stochastic generative model used in the main text, the Cayley trees do not follow a linear relation. Instead, the points fill in an allowed region of phase space, but are otherwise uncorrelated, indicating that α and β are not inherently redundant. Instead, we conclude that the generative model has additional properties that lead to a relation between these two extracted values.**(B)** Examples of scaling fits for the α and β parameters. Cayley tree growth is more complicated than a power-scaling law for both exponents, but a linear fit can be used to estimate the essential behavior.

VI. ESTIMATING THE SIMULATION PARAMETERS FROM THE BRANCHING RATE

Each simulation network has three input parameters: the stretching factor s, the branching factor b and the number of nodes in the network N. To extract the scaling relations, we use quantities that are functions of all three parameters: $A(s, b, N)$ and $R_v(s, b, N)$. Note that the total network length L and the total number of branches B are functions of just two of the parameters: $L = L(s, N$ and $B = B(b, N)$. We can write down close-form expressions for these quantities. First, we write down the expected number of branches after N steps. A branching event occurs with probability b, and after a new branch is added the total number of branches increases by 2. So, the expected number of network branches B after N simulation steps is

$$B(b, N) = 1 + 2bN. \tag{1}$$

This is a good estimate if edge overlaps are allowed in the network structure. However, terminal cells are modeled as networks with no edge overlaps, and this is enforced in the simulation algorithm by rejecting a newly-placed edge that has overlapped with an existing part of the network. In the networks, edge overlaps are more likely to arise from a growing branch tip than a newly-formed branch bud. This results in the final non-overlapping network structures having more branches than expected from the equation 1. We confirm this through simulations in Fig. 6, where we see that the equation 1 estimates the number of branches well in the case of overlapping networks and underestimates the number of branches in networks where non-overlapping networks.

The total length of the network is a function of the stretching parameter s, the number of time steps N, and the length of each additional piece of network added per time step dx. The simulation works by adding a new length dx at every time step and then stretching the whole space by s. In the case $s = 0$ the length is $L(0, N) = L_0 + Ndx$. Otherwise, we calculate the first few terms to set up the general form:

$$\begin{aligned}
L_1 &= L_0(1+s) + dx \\
L_2 &= L_1(1+s) + dx = L_0(1+s)^2 + \big((1+s) + 1\big)dx \\
L_3 &= L_2(1+s) + dx = L_0(1+s)^3 + \big((1+s)^2 + (1+s) + 1\big)dx \\
&\vdots \\
L_N &= L_0(1+s)^N + \big((1+s)^{N-1} + (1+s)^{N-2} + ... + (1+s) + 1\big)dx
\end{aligned} \tag{2}$$

Using the formula $(x^{N-1} + x^{N-2} + ... + x + 1)(x - 1) = x^N - 1$ we arrive at:

$$L(s, N) = L_0(1+s)^N + \frac{(1+s)^N - 1}{s} dx \tag{3}$$

In simulations, we set $dx = 1$ micron. Because each simulation step always consists of one stretching event followed by adding one new segment, the expression for L is exact. Indeed, equation 3 described simulated data well for both overlapping and non-overlapping networks, verified by simulations in Figure 6.

Given the approximate expressions $L(s, N)$ and $B(b, N)$ we can fit a function of the form $L(B)$ to the experimental data by minimizing the residual sum of squares between the curve and the data. Residuals are computed on a log-log scale, otherwise the latest-stage cells dominate, since they are several orders of magnitude larger that the early-stage cells in both L and B. The best-fit parameter set is $s = 0.0014$ and $b = 0.0399$. Confidence intervals are computed using a bootstrap analysis of the data, resampling with replacement. We generate 200 new data sets by repeatedly choosing points uniformly at random from the data. Because the size of the initial data set it 162 points, each resampled data set also has 162 points. The s and b values are computed again through the same fitting procedure, as shown in Figure 7. Because resampling gives an estimate on how the fit estimates vary, they are used to provide confidence intervals on the best-fit values. The point spread in s and b is shown in Figure 7, the 80% confidence intervals are calculated by taking the 10th and 90th percentile values in these distributions, and the result is (0.0013, 0.0017) for s and (0.037, 0.043) for b. This simplified method does not account for the geometric behavior of the self-avoiding networks, and using the full scaling calculations for the convex hull area and the mean void radius is a more robust calculation. Nonetheless, it is interesting that these approximate values agree well with the best-fit values from the main paper result, $s = 0.0007$ and $b = 0.055$.

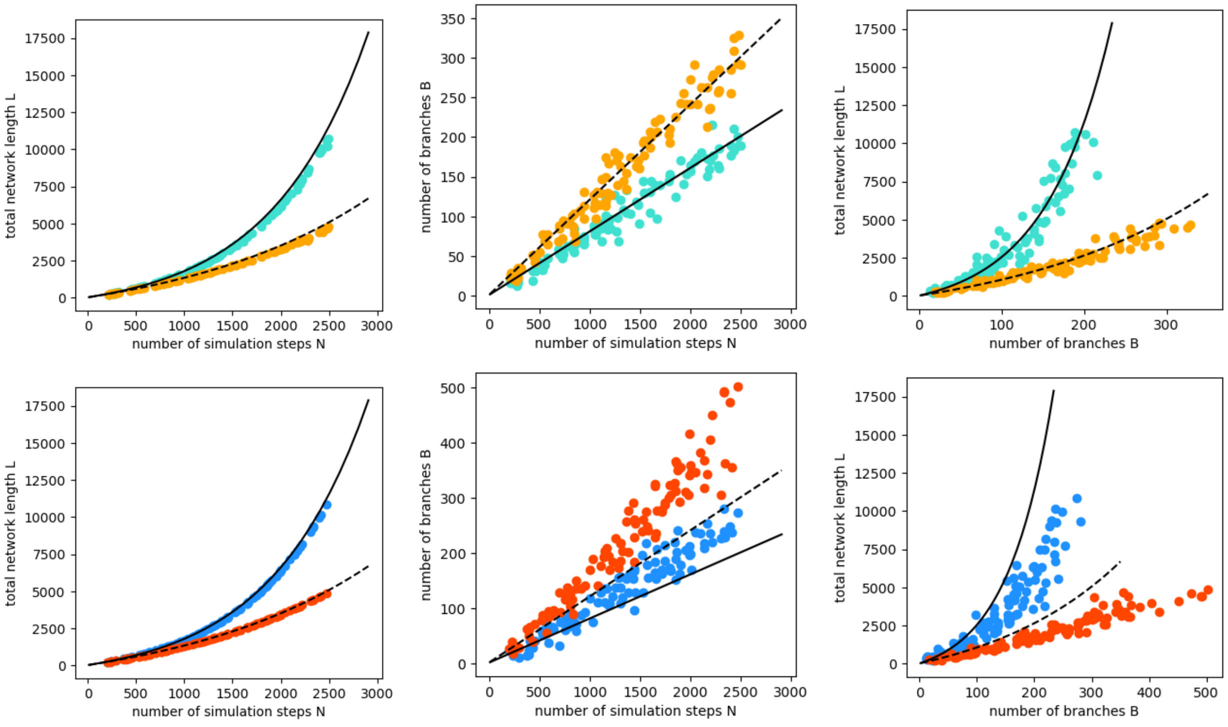

SUPPORTING FIG. 6. A comparison between the derived expected number of branches (equation 1) and the total edge length (equation 3) and these values from simulated networks of different sizes and for two sets of parameters (s, b). The top row is a comparison with simulations where edge overlap is allowed, and in the bottom row the simulations are restricted to be non-overlapping, as in the main text. The blue and turquoise data points and solid lines have parameters set to $s = 0.001$ and $b = 0.04$, while the red and orange data points and dashed lines have $s = 0.0005$ and $b = 0.06$. In both the overlapping and non-overlapping networks, equation 3 describes the data well, up to numeric error. Equation 1 is a good estimate for the overlapping networks but underestimates the number of branches for overlapping networks.

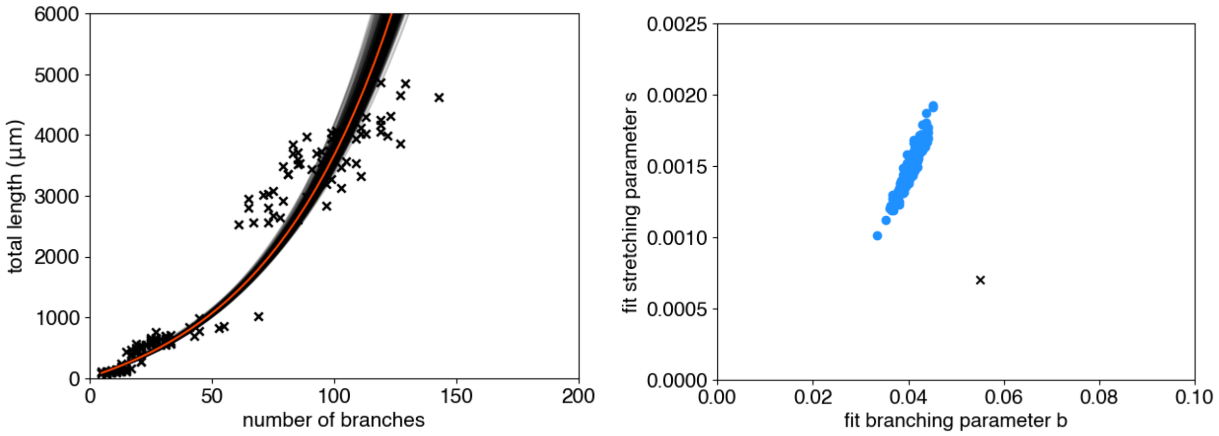

SUPPORTING FIG. 7. (A) The red curve is the global best fit to the data, with $s = 0.0014$ and $b = 0.0399$. The black curves are 200 fits to a bootstrapped subsample of the data. The 80% confidence intervals for s and b are: (0.0013, 0.0017) and (0.037, 0.043). (B) A scatter plot of the extracted s and b values from each subsampled data set. The parameter fitting result from the scaling analysis is $s = 0.0007$ and $b = 0.055$, indicated by the cross.

VII. COMPARING CROSS-SECTIONAL AND LONGITUDINAL DATA SETS

In the branching analysis of the previous section we have used the heat-killed larval data (cross-sectional samples from three time points) as opposed to the time-lapse experiments (Figure 2C and 2D). There are two reasons against using the time-lapse data. First, removing the larva from the food for the imaging experiments disrupts developmental time. We have not quantified the amount of developmental slowing, but we have observed that larvae imaged multiple times spend longer than the expected 5 days in the larval stage before pupation. Second, the intensity of GFP in living larvae is more faint than in heat-killed larvae. We are confident that we capture the full network structure in the measurements for Figure 3F and 3G. However, the traces for Figure 2C and 2D are likely missing network edges, which is the time lapse experiments are used only for measuring internal branches, and not the appearance of new branches.

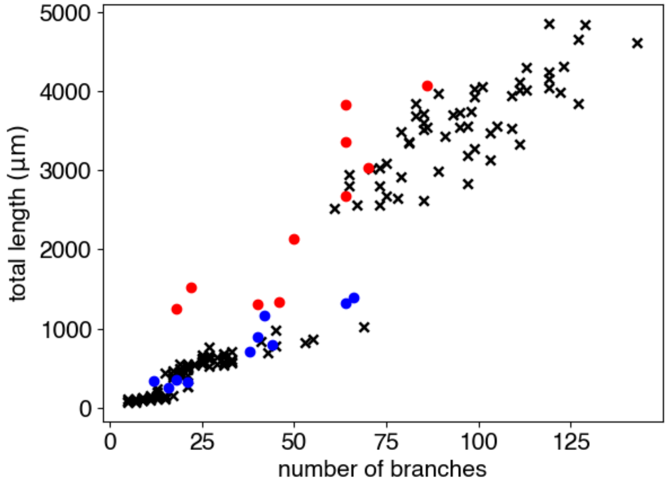

SUPPORTING FIG. 8. The cross-sectional data (black crosses) in comparison with the time-lapse data, where the early time point is shown in blue and the late time point is shown in red. The time-lapse data underestimates both the number of branches and the total length in the later time point.
